# Association between depression and insulintherapy

**DOI:** 10.1192/j.eurpsy.2023.510

**Published:** 2023-07-19

**Authors:** F. Zaouali, A. El Khemiri, F. Boubaker, S. Arfa, B. Zantour, W. Alaya, M. H. Sfar

**Affiliations:** Endocrinology Depatment, Tahar Sfar University Hospital, Mahdia, Tunisia

## Abstract

**Introduction:**

Insulin is the basic medical therapy to manage type 1 diabetes and is also a cornerstone of treatment of type 2 diabetes as insulinopenia belongs to its natural history. However, insulintherapy is associated with many challenges especially psychological difficulties such as patient’s acceptance and compliance, which may lead to metabolic and psychological disorders.

**Objectives:**

The aim of our study was to determine the association between insulintherapy and depression.

**Methods:**

A cross sectional analytic study was conducted from October 2019 to October 2020 among a group of diabetic patients followed in the Endocrinology Department of Taher Sfar University Hospital in Mahdia, Tunisia. “DSM-V diagnosis criteria for depression screening” and “Hamilton score scale” were used to evaluate the severity of depression.

**Results:**

A total of 260 patients were recruited in our study. The mean age was of 57.36±15.4 years with extremities ranging from 20 to 91 years. The sex ratio M/F was situated at 0.59. The mean diabetes duration was of 10.92 years. The majority of patients had type 2 diabetes (92.3%). The micro vascular long-term complications of diabetes were the most frequent (67.7%): neuropathy (39%), retinopathy (37%) and nephropathy (24%). According to the “DSM-V diagnosis criteria”, 15% of the study population suffered from a Major Depressive disorder (MDD). Hamilton score scale showed that thirty-eight patients had severe depression symptoms (14.6%). Insulintherapy was associated with MDD and depression severity (19.1% vs 10.1% ; p=0,041 and 20% vs 8.4% ; p*<10^–3^*).

**Conclusions:**

Diabetic patients treated with insulin seem to be exposed to severe depressive syndromes. Once insulin initiated, doctors should be careful at the psychological aspects and the burden of this decision and use in consequence appropriate tools to screen depressive symptoms and anxiety. The role of family doctor is crucial providing early psychological support and preventing complications associated with depression especially poor glycemic control.

**Disclosure of Interest:**

None Declared

